# Sinapic Acid Ameliorates the Progression of Streptozotocin (STZ)-Induced Diabetic Nephropathy in Rats *via* NRF2/HO-1 Mediated Pathways

**DOI:** 10.3389/fphar.2020.01119

**Published:** 2020-07-23

**Authors:** Ahmed L. Alaofi

**Affiliations:** Department of Pharmaceutics, College of Pharmacy, King Saud University, Riyadh, Saudi Arabia

**Keywords:** sinapic acid, diabetic nephropathy, apoptosis, inflammation, NRF2/HO-1 pathways

## Abstract

Diabetic neuropathy (DN) is a complicated inauspicious outcome of diabetes, like other abnormalities of diabetes the cause of DN is still vague and it may be the result of various pathological conditions leading up to end-stage renal failure. The present study examines the efficacy of sinapic acid (SA) in streptozotocin (STZ)-induced DN nephropathy and the linked pathway. Twenty-four rats were equally divided randomly into four categories: Normal control (NC), STZ, STZ + SA 20 mg/kg bw, and STZ + SA 40 mg/kg bw. After 8 weeks they were evaluated for ratio of renal index, the fasting blood glucose (FBG), blood urea nitrogen (BUN), 24 h urea protein, serum creatinine (SCr), reduced glutathione peroxidase (GPx), superoxide dismutase (SOD), lipid peroxidation (MDA), tumor necrosis factor α (TNFα), interleukin (IL)-6, as well as lipid profile total cholesterol (TC), total triglycerides (TG), very low density lipoprotein (VLDL), low density lipoprotein (LDL), and high density lipoprotein (HDL) levels. Additionally, histomorphology and ultrastructure of the kidneys were also assessed. Protein expression levels of transforming growth factor-β1 (TGF-β1), nuclear factor erythroid 2-related factor 2 (Nrf2), heme oxygenase-1 (HO-1), IκBα protein (IkBα), anti-apoptotic protein BCl2, nuclear factor kappa B (NF-kB), and Bax were examined. We observed that SA 20 mg/kg bw and 40 mg/kg bw pretreatment significantly and dose-dependently upregulated the protein expression of HO-1, Nrf2, IKBα, and Bcl-2 but downregulated the protein expression of NF-κB, proposing that the nephroprotective mechanism of SA is due to its antioxidant and anti-inflammatory activity; SA prevents the release of cytokines and inflammatory markers (TNFα and IL-6), upregulates antioxidant defense enzymes, and reduces lipid peroxidation, as well as nitric oxide, and anti-apoptotic activity, which may be influenced by the regulation of TNF-α, IL-6, Bcl-2, NF-kB, and BaX *via* the Nrf2/HO-1 pathway in STZ induced DN. Thus, our results suggest that SA ameliorates the development of STZ-induced DN in rats *via* NRF2/HO-1 mediated pathways. Further comprehensive studies are required for complete elucidation of the fundamental mechanisms.

## Highlights

- STZ induced diabetic rats displayed renal tubular damage and glomerular injury as evident by increased FBG, BUN, 24 h urea protein and increase in renal hypertrophy index.
- SA pretreatment prevents renal tubular oxidative stress, inflammation, and apoptosis in diabetic rats.
- SA pretreatment inhibits apoptosis in renal tubules by downregulating apoptotic protein caspase 3, Bax and upregulating anti-apoptotic protein Bcl-2.
- SA pretreatment modulates the Nrf2/HO-1 pathway to upregulate and anti-oxidant defense enzymes SOD, GPX, and catalase in diabetic rats.

## Introduction

Diabetes mellitus (DM) is a diversely complicated disease of insulin dysfunction often associated with dreadful repercussion by hyperglycemia, hypoinsulinemia, and glycosuria. Diabetes mellitus (DM) encapsulates a variety of severe metabolic diseases distinguished by hyperglycemia, hypoinsulinemia, and glycosuria. DM is a multifaceted syndrome comprising insulin impairment along with glucose steady state and lipid metabolism abnormalities. Moreover, the parallel high level of blood glucose and (or) low insulin conditions, ultimately, might have serious injurious consequences on the body physiology (Hoybergs et al., 2008). Therefore, DM is a major threat to public health and its prevalence is increasing as this disease can cause long-lasting impairments of physiological functions and failures in organs with widespread impediments (Meo et al., 2015; Satheesh et al., 2020). For instance, 40% of end stage renal disease (ESRD) cases in diabetic patients because of diabetic nephropathy (Kundu et al., 2020). Due to hyperglycemia, there might be numerous toxicity effects that can occur in diabetic patients at the physiological and metabolic levels. This would lead to unpleasant consequences, as a result of hyperglycemia, such as infections, promoting cancers, proteins modification, and many other concerns. Therefore, the management of hyperglycemia remains the primary goal in DN therapy (Giri et al., 2018). The oral hypoglycemic agents used in the management therapies for DM must improve the pathophysiology by increasing insulin secretion and/or sensitivity, as well as inhibit hepatic glucose production *via* the downregulation of gluconeogenesis (DeFronzo et al., 1992; Wilson et al., 2011; Nithya and Subramanian, 2015; Nithya et al., 2017). The oral hypoglycemic agents used to alleviate DN are adipogenic (Klein et al., 2007), and synthetic oral hypoglycemic therapies are closely associated with various toxicities (Spiller and Sawyer, 2006). DM can be managed by oral hypoglycemic agents and insulin, but these approaches fail to clinically reinstate normoglycemia in most patients (Scheen, 1997). The World Health Organization (WHO) endorses the use of herbs or natural products for the management of DM as trustworthy with few or no side effects. In fact, these natural products are deliberated as good contenders for oral therapy (Shokeen et al., 2008). Prolonged hyperglycemia persuades the accumulation of reactive oxygen species (ROS) (Nishikawa et al., 2000), and excessive ROS production inflicts tissue injuries (Giacco and Brownlee, 2010). Various factors contribute to the progression of DN, including hyperglycemia, glycosuria, hypertension, ketoacidosis, coronary heart disease, obesity, and advancing age, but the exact mechanism remains elusive (Brownlee, 2005). Notably, the progression of DN is linked with oxidative stress and inflammation (Wee, 1991). Moreover, ROS is critically involved in the progression of DN (Stenvinkel et al., 2018). The transcription factor, nuclear factor erythroid 2-like 2 (Nrf2), is a major mediator controlling redox enzymes defense systems in reaction to oxidative stress. Nrf2 is seized in the cytoplasm *via* its binding to Kelch-like ECH-associated protein 1 (Keap1) (Itoh et al., 1999b). Oxidative stress encourages translocation of Nrf2 to the nucleus, where it upregulates the antioxidant defense enzymes and heme oxygenase-1 (HO-1) (Nguyen et al., 2003; Ray et al., 2012). The enhancement of ROS levels, including superoxides and hydrogen peroxide, in DN has been reported (Ricardo et al., 1994). Nrf2 impairment in DN causes accumulation of peroxide radicals inflicting renal tissues injuries (Nezu et al., 2017). Hence, the improvement (i.e. upregulation) of Nrf2/HO-1 has been gaining popularity as a therapeutic approach. Several Nrf2 activating natural polyphenols have been reported to ameliorate DN in patients (Sun et al., 2017; Xie et al., 2018; Zhang et al., 2018). SA (3,5-dimethoxy-4-hydroxycinnamic acid), that can be found in berries, vegetables, cereals, and oilseed crops (Andreasen et al., 2001), is naturally phenolic compound that can be found in the plant such as fruits, vegetables, cereal grains, and oilseed crops. The list of the medicational effects of SA covers different pharmacological activities such as possesses antioxidant, antidiabetic, anti-inflammatory, anticancer, antimicrobial, antihypertensive, and anxiolytic activities (Nićiforović and Abramovič, 2014; Karthik, 2014; Chen, 2016; Srivastava et al., 2018). SA has a potent chemoprotective effect due to its free radical-scavenging activity that protects against tissue injury (Sharma et al., 2015; Chen, 2016; Chen et al., 2016; Silambarasan et al., 2016). Sinapic acid significantly decrease lipid peroxidation marker TBARS and increases the antioxidants markers like SOD, CAT, GPx, and GSH in serum, liver, and kidney in diabetic rats (Kanchana et al., 2011). Although effect of SA on the biochemical markers (such as blood urea, serum creatinine, uric acid, and FBG) have been investigated previously (DeFronzo et al., 1992; Kanchana et al., 2011; Wilson et al., 2011; Nithya and Subramanian, 2015; Nithya et al., 2017), we here investigating the effect of SA on the gene expression of SOD, GPx, and catalase and the signaling pathway of Nrf2/HO-1 in the STZ-induced DN rats. Thus, high potential of sinapic acid pharmacological activities in diabetic models has instigated us to postulate that SA upregulates Nrf2 nuclear translocation and HO-1 expression. Furthermore, we anticipated that SA activates the Nrf2/HO-1 antioxidant pathway in STZ-induced DN rats.

## Materials and Methods

### Chemicals

STZ, SA, GPx, sodium azide, and thiobarbituric acid were obtained from the same vendor (Sigma Chemicals Co, St. Louis, MO, USA). Antibodies against Nrf2, NF-kB (p65), HO-1, HRP-conjugated secondary antibodies, and β-actin were obtained from Santa Cruz Biotechnology vendor (Dallas, TX, USA). The total protein and NE-PER cytoplasmic and nuclear extraction kit was acquired from Thermo Fisher Scientific (Waltham, MA, USA). Rat TNF-α and IL-6 ELISA kits were procured from R&D Systems, Inc. (Minneapolis, MN, USA).

### Animals

The male wistar rats, of 231–249 g, used in this study were obtained from the Central Animal Facility in King Saud University (CAF-KSU). The animals were kept, fed, and handled as the following: each plastic cage has n = 6 in a 12 h light/dark cycle at controlled temperature of 25 ± 2°C, a standard pellet diet and water *ad libitum* were fed to the rats, and all handling of the animals were correspondingly with Guide for the Care and Use of Laboratory Animals by the Animal Care Center. The Research Ethics Committee of the College of Pharmacy, King Saud University (Riyadh, Saudi Arabia, KSU‐SE‐20‐4) approved this study.

### Induction of Diabetes

The 12-h fasting rats were subjected to diabetes induction using STZ with a dose of 60 mg/kg in a buffer of citric acid at pH 4.5. The route of STZ administration was intraperitoneally (i.p.) (Cheng et al., 2013). After 1 h of the STZ injection, the animals were fed *ad libitum* and kept under observation for approximately 48 h. The rats were then fasted 12 h and FBG levels were examined 72 h of STZ treatment. Animals exhibiting FBG >250 mg/dl were deliberated diabetic animals.

### Experimental Design

The animals were arbitrarily separated into four categories. Group I consisted of six normal rats. The STZ-induced DN rats (Groups II–IV) were grouped into three groups (n = 6) at random. Group II served as the DN control and was administered 2 ml of normal saline per day. Groups III and IV were administered SA (20 and 40 mg/kg•bw/d, respectively). Measurements of body weight, blood glucose, and food and water consumption were taken, and physical examinations were conducted at regular intervals. Blood was collected for serum separation. Fasting blood glucose levels were assessed to confirm the anti-hyperglycemic properties of SA.

### Preparation of Renal Tissue Homogenate

All rat groups (Group I, Group II, Group III, and Group IV) were subjected to renal tissue harvesting perfused in ice-cold saline, homogenized in 100 mM Tris-HCl buffer (pH 7.4) with T 25 Digital ULTRA-TURRAX and centrifuged at 10,000 rpm for 10 min at 4°C. The upper layer was extracted and further used for evaluation of antioxidant enzymes and for the estimation of lipid peroxidation (MDA), renal tissue was homogenized in 1.15% KCl solution to obtain a 10% (w/v) homogenate. Total protein levels were assessed (Lowry et al., 1951). Bovine serum albumin (BSA) was used as a reference.

### Assessment of Renal Dysfunction

To assess the renal dysfunction, the levels of Serum FBG, BUN, 24 h urea protein, and Scr were estimated as described previously (Tikoo et al., 2007a; Tikoo et al., 2007b).

### Oxidative and Antioxidant Indices

To estimate the oxidative stress condition, MDA levels (the most studied final product of LPO) was evaluated using thiobarbituric acid reactive substances method as described by Ohkawa et al. (1979). The generated pink/red-colored product was measured at an absorbance of 532 nm (using spectrophotometer) against the MDA standard curve to determine the level (Niehaus and Samuelsson, 1968). Nitric oxide (NO) was estimated according to the method described by Bryan and Grisham et al. (Bryan and Grisham, 2007). The tissue level of nitrite/nitrate was measured by ELISA kit using the Griess reaction, the absorbance was measured with a microreader, and the nitrite and nitrate concentration of homogenate was examined by comparison with the nitrite/nitrate standard reference curve. Superoxide dismutase (SOD) activity was examined as per the method by Sun et al. (1988). SOD assessment was based on the production of superoxide radicals generated by xanthine and xanthine oxidase, that react with 2 - (4- iodophynyl) - 3 (4 - nitophenol) - 5 -phenyltetra-zolium chloride to form a red formazan dye. The activity of catalase (CAT) was assessed following the method described by Aebi (1974). Briefly, the measurement of CAT activity was done by incubating the homogenate in H_2_O_2_ substrate; then the enzymatic reaction stopped by adding 1 ml of ammonium molybdate. The intensity of the yellow complex formed by molybdate and H_2_O_2_ was measured at 405 nm. Glutathione peroxidase (GPx) activity was assessed following the method, with slight modifications, described by Flohé and Günzler (1984). GPx activity was measured by suspending 1 mg of protein from the homogenate in 1.6 ml of 50 mM phosphate buffer (pH 7.0), contained 0.2 mM NADPH, 1 mM GSH, and 1 UI/ml glutathione reductase; and the mixture was incubated at room temperature for 5 min. Then 100 *μ*l of 0.25 mM H_2_O_2_ was added to the mixture and initial (immediate) and final readings (after 5 min) were taken at 340 nm using spectrophotometer and assessed in the homogenized renal tissue.

### Cytokine and Inflammatory Marker

Assessing the level TNF-α and IL-6 markers were performed in the renal tissue homogenates by ELISA kits obtained from R&D System according to manufacturer protocol. The wavelength measurement of the markers’ absorbance was 450 nm.

### Preparation of Total Protein, Cytosol, and Nuclear Protein

Renal tissue cytosol and nuclear protein were extracted using an NE-PER kit (Thermo Fisher Scientific). Immunoblots were executed as per the procedures of Towbin et al. (1979). The protein of 25 µg was transferred to polyvinylidene difluoride (PVDF) membrane and blocked in a blocking buffer (4% skim milk in TBS of 1% Tween 20). Then, the membrane was incubated overnight (4°C) with antibodies against NRF2, HO-1, NF-κB (p65), and β-actin. After repeated washing steps with 1% Tween TBS and TBS, the membrane was incubated with secondary antibodies for 2 h (room temperature). Bands visualization were done using Luminata™ Western Chemiluminescent HRP Substrates (Millipore, Billerica, MA, USA) and densitometric analysis of the immunoblots (LI-COR C-Di-Git Blot Scanners, Lincoln, NE, USA).

### Histological Analysis

Fixation of the renal tissues were done as the following: 10% formalin, then dehydrated with gradient alcohol and soaked in paraffin blocks. Afterward, the tissues were sliced into sections of 4 μm and stained with hematoxylin and eosin (H&E) dye and examined under a light microscope.

### Statistical Analysis

The statistical significance was carried out using one-way-analysis of variance (ANOVA) and *post-hoc* with Dennett’s test to determine the significant differences. All data were reported here with arithmetic means ± SEM.

## Results

Our results showed 85% of the rats have type-II diabetes which was induced by STZ. These rats exhibited signs of glycosuria, polyuria, hyperglycemia, body weight loss, and water uptake enhancement. The loss of body weight (BW) in rats in comparison to normal control (NC) was considered as a sign of elevated blood sugar. Therefore, changes in the initial and final BW of the NC and STZ induced DN were observed and recorded (**Table 1**). SA oral administration (20 and 40 mg/kg B.W.) exhibited significant nephroprotection from considerable weight loss (p < 0.05). The renal weight and kidney hypertrophy index in STZ-induced DN rats were significantly increased in comparison to those in the NC group. SA oral administration (20 and 40 mg/kg B.W.) to STZ-induced diabetic rats significantly ameliorated the kidney hypertrophy index (**Table 1**).

**Table 1:** Initial and final body weights of rats (n = 6).

Animals	Initial body weight (g)	Final body weight (g)	% gain	Renal weight (g)	Renal hypertrophy index
**Normal**	247.00 ± 3.50	294.83 ± 3.59	16.22	1.25 ± 0.03	0.42 ± 0.01
**STZ-induced DN**	240.33 ± 2.22	170.33 ± 2.12	−41.10	1.49 ± 0.04	0.87 ± 0.02
**STZ+SA 20 mg/kg bw**	243.17 ± 3.93	195.00 ± 2.85	−24.70	1.30 ± 0.03	0.66 ± 0.02
**STZ+SA 40 mg/kg bw**	250.50 ± 3.49	212.67 ± 1.15	−17.79	1.25 ± 0.02	0.58 ± 0.01

### Biochemical Findings

For assessing hyperglycemia, FBG levels were measured after 72 h of administering STZ to the rats; 87.5% of the rats were found to be diabetic with an average FBG level of 533.67 ± 3.90 mg/dl. The STZ-induced DN rats (Groups II–IV) were arbitrarily divided into three groups (n = 6). After 8 weeks of SA oral administration (20 and 40 mg/kg B.W.), these rats exhibited a significant dose-dependent reduction in FBG levels. The SA (20 and 40 mg/kg B.W.)-administered diabetic rats exhibited amelioration of hyperglycemia in a dose dependent manner, with average FBG levels of 359.40 ± 5.11 mg/dl (37.9%) for a 20 mg/kg SA dose and 294.96 ± 4.76 mg/dl (49.04%) for a 40 mg/kg SA dose. However, the average FBG level in non-treated diabetic rats was 578.83 ± 3.89 mg/dl (**Table 2**). Normal control rats exhibited FBG levels of 91.17 ± 2.37 at 72 h and 90.17 ± 1.92 after 8 weeks, indicating no change. We have noticed significant enhancement in the level of BUN (215.80%), 24 h urea albumin protein (208.75%), LDH (222.67%), and creatinine (151.3%) in STZ-induced diabetic rats. Oral administration of 20 and 40 mg/kg SA to diabetic rats showed significant dose-dependent reductions in BUN (35.21 and 48.88%, respectively), 24 h urea protein (33.83 and 41.57%, respectively), LDH (25.79 and 56.73%, respectively), and creatinine (28.81 and 41.47%, respectively) levels (**Table 3**). These significant reductions indicate the amelioration of STZ-induced DN in experimental rats.

**Table 2:** Assessment of FBG levels in STZ-induced diabetic rats.

Animals	72 H (mg/dl)	8 Weeks (mg/dl)
**Normal**	91.17 ± 2.37	90.17 ± 1.92
**STZ-induced DN**	533.67 ± 3.90	578.83 ± 8.27
**STZ+SA 20 mg/kg bw**	559.0 ± 7.34^*#^	359.4 ± 5.11^*#^
**STZ+SA 40 mg/kg bw**	570 ± 0.03^*#^	294.97 ± 4.76^*#^

The mean ± SEM of six animals in each group were calculated. “*” Indicates significant differences to the STZ-induced diabetic group (P value < 0.05); “#” indicates significant differences to the normal control.

**Table 3:** Assessment of renal dysfunction levels in STZ-induced diabetic rats.

Animals	Creatinine (mg/dl)	Blood urea nitrogen (mg/dl)	LDH(nmol/l)	Total albumin 24 H (mg/day)
**Normal**	0.48 ± 0.02	20.02 ± 0.87	64.04 ± 1.47	1.34 ± 0.15
**STZ-induced DN**	1.21 ± 0.05	63.22 ± 1.93	206.64 ± 4.57	2.80 ± 0.36
**STZ+SA 20 mg/kg bw**	0.86 ± 0.03^*#^	40.96 ± 1.18^*#^	153.35 ± 3.59^*#^	1.85 ± 0.07^*#^
**STZ+SA 40 mg/kg bw**	0.71 ± 0.03^*#^	32.32 ± 1.14^*#^	89.42 ± 2.29^*#^	1.64 ± 0.10^*#^

The mean ± SEM of six animals in each group were calculated. “*” Indicates significant differences to the STZ-induced diabetic group (P value < 0.05); “#” indicates significant differences to the normal control.

### Serum Lipid Profile

The serum lipid levels of TC, TG, VLDL, and LDL were evaluated in the treated STZ-DN rats with 20 and 40 mg/kg SA (**Table 4**). As represented in the **Table 4**, the levels of serum TC, TG, VLDL, and LDL were significantly augmented in diabetic rats at 204.93, 202.55, 202.69, and 661.07%, respectively, when compared to the normal ones. However, the levels of serum HDL were significantly reduced by 23.97% in DN rats, in comparison to normal ones. Also, pretreatment of 20 and 40 mg/kg SA can increase 48.63 and 32.21%. The pretreatment with a 20 mg of SA can significantly reduce TC and TG levels to 35.24 and 42.90% in comparison to STZ induced DN rats. For pretreatment with 40 mg of SA, the TC and TG levels showed 51.97 and 53.19% reduction in comparison to STZ induced DN rats. Both 20 and 40 mg pretreatments showed significant and dose dependent reduction for TC and TG. Also, the levels of VLDL and LDL in pretreatment rats were reduced significantly and, in a dose, dependent manner to 42.90 and 49.69% with pretreatment 20 mg of SA and to 53.19 and 68.28% with pretreatment 40 mg of SA, respectively.

**Table 4:** Assessment of the serum lipid profiles in STZ-induced diabetic rats.

Animals	TC(mmol/L)	TG(mmol/L)	VLD(mmol/L)	LDL(mmol/L)	HDL (mmol/L)
**Normal**	65.75 ± 3.54	63.68 ± 8.67	12.74 ± 0.53	17.76 ± 3.94	35.25 ± 2.51
**STZ-induced DN**	200.50 ± 8.21	192.67 ± 3.55	38.53 ± 0.71	135.17 ± 8.70	26.80 ± 1.44
**STZ+SA 20 mg/kg bw**	129.83 ± 7.55^*#^	110.0 ± 2.62^*#^	22.0 ± 0.52^*#^	68.0 ± 6.59^*#^	39.83 ± 1.93^*^
**STZ+SA 40 mg/kg bw**	96.33 ± 3.52^*#^	90.18 ± 2.04^*#^	18.04 ± 0.41^*#^	42.86 ± 3.48^*#^	35.43 ± 2.19^*^

The mean ± SEM of six animals in each group were calculated. “*” Indicates significant differences to the STZ-induced diabetic group (P value < 0.05); “#” indicates significant differences to the normal control.

### Antioxidant Effects of SA

As illustrated in **Table 5**, GPx, SOD, and catalase activities were significantly reduced to 57.34, 67.43, and 57.66%, respectively, due to depletion of antioxidant enzymes in STZ-induced diabetic rats compared to normal rats. The oral administration of 20 and 40 mg/kg SA significantly enhanced GPx, SOD, and catalase activity to 46.073 and 74.52%, 29.47 and 60.44%, and 77.34 and 108.49%, respectively, indicating the restoration of antioxidant defense enzymes.

**Table 5:** Antioxidant effects of sinapic acid in STZ-induced diabetic rats.

Animals	CAT(U/mg protein)	GPX(U/mg protein)	SOD(U/mg protein)
**Normal**	12.34 ± 0.35	522.86 ± 8.67	78.87 ± 2.82
**STZ-induced DN**	5.22 ± 0.14	203.01 ± 6.89	25.68 ± 1.59
**STZ+SA 20 mg/kg bw**	9.26 ± 0.31^*#^	325.75 ± 5.23^*#^	33.25 ± 1.28^*#^
**STZ+SA 40 mg/kg bw**	10.88 ± 0.41^*#^	389.21 ± 10.85^*#^	41.21 ± 2.04^*#^

The mean ± SEM of six animals in each group were calculated. “*” Indicates significant differences to the STZ-induced diabetic group (P value < 0.05); “#” indicates significant differences to the normal control.

### Oxidative Stress Markers MDA and NO

The kidneys of STZ-treated diabetic rats exhibited significant enhancements in MDA levels (140.42%; P < 0.05; **Table 6**) and NO content (78.98%; P < 0.05; **Table 6**) compared to those of the normal control rats. However, the oral administration of 20 and 40 mg/kg SA significantly reduced MDA and NO2 activity to 33.70 and 46.07% (P < 0.05 or P < 0.05) and 15.88 and 33.79% (P < 0.05 or P < 0.05), respectively, indicating the abolition of oxidative stress.

**Table 6:** Oxidative stress marker (MDA) in STZ-induced diabetic rats.

**Animals**	**NO_2_** **(nmol/mg protein)**	**MDA** **(ng/mg protein)**
**Normal**	29.90 ± 1.01	10.08 ± 0.71
**STZ-induced DN**	53.52 ± 1.61	24.25 ± 1.22
**STZ+SA 20 mg/kg bw**	45.01 ± 1.56^*#^	16.08 ± 0.32^*#^
**STZ+SA 40 mg/kg bw**	35.43 ± 1.02^*#^	13.08 ± 032^*#^

The mean ± SEM of six animals in each group were calculated. “*” Indicates significant differences to the STZ-induced diabetic group (P value < 0.05); “#” indicates significant differences to the normal control.

### Inflammatory Markers and Cytokines

The results validated that TFN-α and IL-6 was significantly enhanced in the STZ-induced diabetic kidneys at 265.66, and 248%, respectively (all P < 0.05) compared to the control rats. Additionally, 20 and 40 mg/kg SA pretreatment significantly and dose-dependently downregulated these effects (P < 0.05 or P < 0.05; **Table 7**) to 29.69 and 50.38%, respectively, for TFN-α; 30.16 and 43.68%, respectively, for IL-6 compared to those in the STZ-induced diabetic control rats.

**Table 7:** Assessment of cytokine and inflammatory markers in STZ-induced diabetic rats.

Animals	TNF-α(pg/mg protein)	IL-6(pg/mg protein)
**Normal**	28.67 ± 1.68	33.59 ± 1.38
**STZ-induced DN**	104.83 ± 1.95	83.33 ± 4.41
**STZ+SA 20 mg/kg bw**	73.71 ± 0.1.73^*#^	58.1+9 ± 2.69^*#^
**STZ+SA 40 mg/kg bw**	52.01 ± 1.54^*#^	46.93 ± 1.79^*#^

The mean ± SEM of six animals in each group were calculated. “*” Indicates significant differences to the STZ-induced diabetic group (P value < 0.05); “#” indicates significant differences to the normal control.

### Western Blot

**Figure 1:**
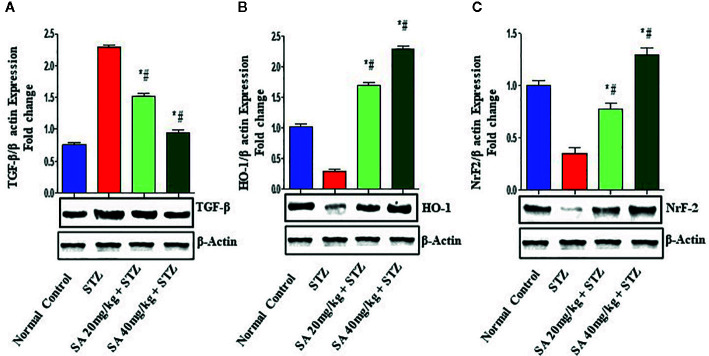
Effect of sinapic acid on the protein expression of **(A)**. Transforming growth factor-β1 (TGF-β1), **(B)** heme oxygenase-1 (HO-1), **(C)** nuclear factor erythroid 2-related factor 2 (Nrf2). The reported results are presented as the mean ± SEM of six animals per group. “*” Indicates significant differences to the STZ-induced diabetic group (P < 0.05); “#” indicates significant differences to the normal control.

The Nrf2/HO-1 signaling pathway plays a crucial role in antioxidant reactions (Wang J. et al., 2015; Shen et al., 2017). To examine whether the pathway is associated with the protective effects of 20 and 40 mg/kg SA on STZ-induced oxidative nephrotoxicity, the expression of Nrf2 and HO-1 were examined (**Figure 1**). Overall, 20 and 40 mg/kg SA pretreatment significantly and dose-dependently upregulated the expressions of Nrf2, HO-1, IkBα, and Bcl-2 but downregulated NF-κB, caspase 3, and Bax protein expression in renal tissue, in comparison to those in STZ-induced diabetic control rats (P < 0.05).

### Histopathological Findings

**Figure 2:**
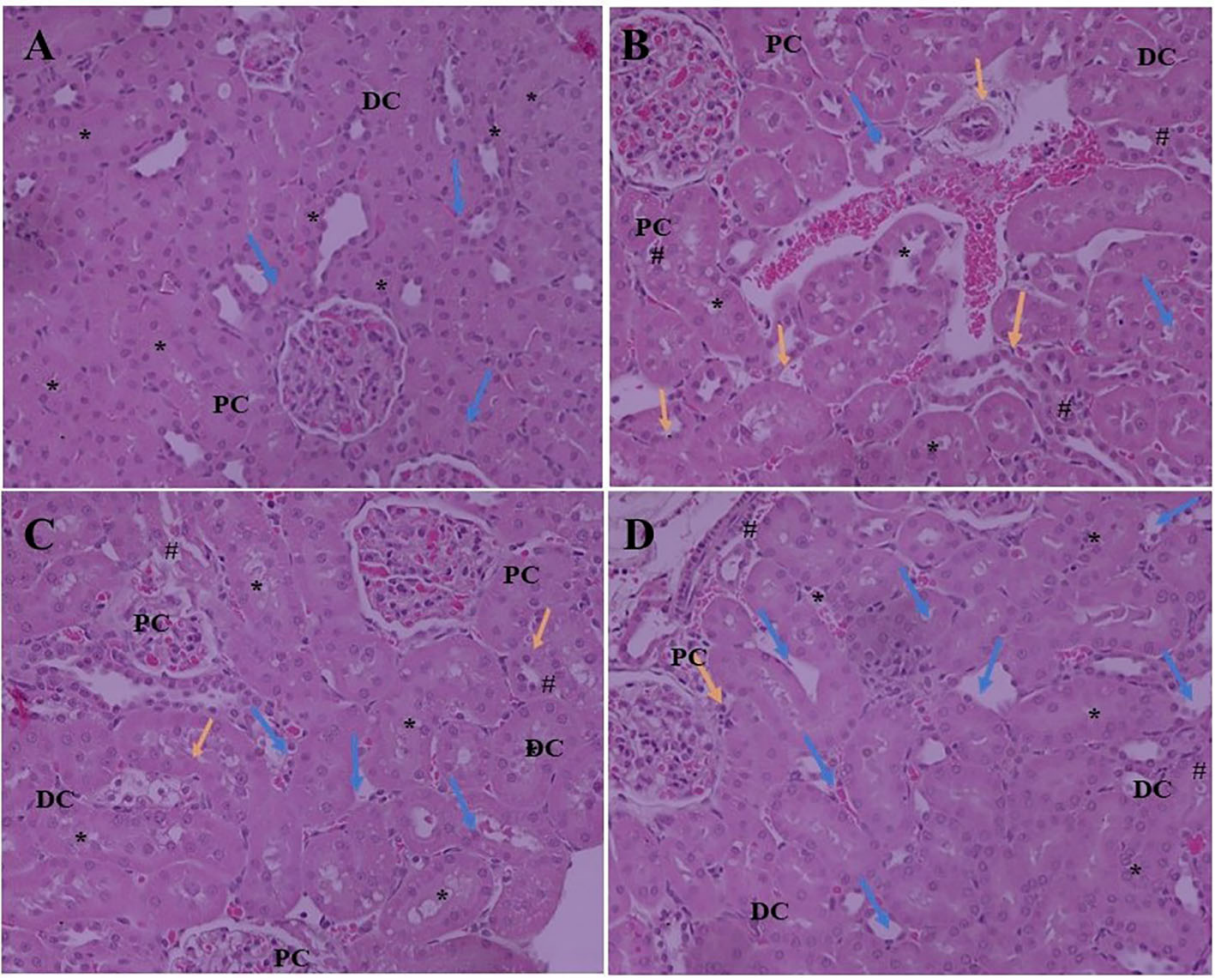
Effect of SA (20 and 40 mg/kg/day) on structural histological features of STZ-induced diabetic nephropathy (H&E). **(A)** Renal tissue of slides of normal control rats exhibits the normal architecture of the glomerulus capillaries, with both proximal convoluted tubules (PC) and distal convoluted tubules (DC) exhibiting a normal tubular epithelium (*). **(B)** Renal tissue of slides of STZ induced diabetic rats the altered architecture glomerular capillaries are widened, irregular, and attached to the Bowman’s capsule (Blue arrow) and exhibiting nodular sclerosis and necrosis (Orange arrow). The tubular epithelium is also altered (*). **(C)** Renal tissue of slides of STZ induced diabetic rats after treatment with 20 mg SA; the altered features are moderately improved compared to normal control rats. The glomerular capillaries retain their normal size and appearance (Blue arrow), but the mesangial cell number is still relatively higher than that of normal rats (#) and the tubular epithelium is reduced (*). **(D)** Renal tissue of slides of STZ induced diabetic rats after treatment with 40 mg SA; the histological features impressively restored towards normalcy; there is a nearly normal structure of glomerular capillaries and Bowman capsule (Blue arrow) and tubular epithelium (*) and exhibit minimal nodular sclerosis and necrosis (Orange arrow).

Expansion of glomerular mesangium a key feature of DN, that is encouraged by mesangial cell proliferation and deposition of extracellular matrices as depicted in **Figure 2**. Normal rats exhibit normal architecture of cortical glomerulus and mesangium without extracellular matrix deposition. SA acid 20 and 40 mg/kg administration significantly restored the cortical glomerulus and mesangium with less deposition of extracellular matrix as compared to DN rats.

## Discussion

DM is often associated with the lethal impediment of DN, which is the key prevalent cause of terminal-stage kidney failure. Overall, 15–25% of type 1 and 30–40% of type 2 patients with DM suffer from DN (Gheith et al., 2016). In this study, STZ-induced type 1 diabetes was examined to understand the DN (Firat et al., 2012; Raish et al., 2016). STZ is the most commonly used agent for inducing type 1 DM because it can trigger specific necrosis of β islets of pancreatic cells that causes degranulation and reduces their ability to discharge insulin, thus, instigating hyperglycemia and nephropathy (Bidani et al., 2007). STZ-induced diabetes is frequently linked with a significant decline in BW and kidney hypertrophy index due to hyperglycemia, hypoinsulinemia, and the loss of muscles proteins (Cheng et al., 2013; Mestry et al., 2017). The body weight and kidney hypertrophy indices of STZ-induced DN gradually decreased and treatment with SA (20 and 40 mg/kg•bw/d) increased BW significantly, thus preventing muscle tissue loss due to hyperglycemia.

In the present study, the STZ-induced diabetic rats experienced damage to kidney functions, which were identified through the substantial elevation of BUN, 24 h urea, and creatinine levels and instability in renal histomorphological architecture, that was obvious by tubular necrosis, grave glomerular mobbing, and intertubular hemorrhage. These results are consistent with those of previous studies (Raish et al., 2016; Sun et al., 2017; Zhang et al., 2018). Pretreatment with SA (20 and 40 mg/kg•bw/d) prevented the progression of DN by significantly reducing kidney function markers, such as 24 h urea protein, BUN, and creatinine, and alleviating ultrastructural changes linked with the amelioration of kidney injuries due to oxidative stress, fibrosis, and inflammation (Tan et al., 2007). Hyperglycemia induced oxidative and osmotic stress causes DN (Dunlop, 2000; Sugimoto et al., 2001). Several *in vitro* studies have illustrated that the accumulation of ROS under hyperglycemic conditions leads to renal injuries (Debnam and Unwin, 1996; Fridlyand and Philipson, 2006; Jeong et al., 2009). ROS accumulation substantially increased in cortical glomerulus cells, comprising endothelial, mesangial, and tubular epithelial cells under hyperglycemic conditions (Kiritoshi et al., 2003). The accumulation and overexpression of TGF-β in kidneys may lead to the development of renal hypertrophy. Protein synthesis rate increase and kidney extracellular organelle degradation reduction may also lead to renal hypertrophy. SA pretreatment abridged kidney hypertrophy indices thus, validating the reversal of renal hypertrophy in STZ-induced DN rats. Changes in oxidative stress markers (MDA) and antioxidant defense enzymes indicate oxidative stress in the cells (Anjaneyulu and Chopra, 2004; Pan et al., 2010). SA treatment significantly inhibited both nitric oxide and lipid peroxidation in STZ induced DN rats. it was reported that SA caused a reduction in renal NO levels in injured rat kidneys (Silambarasan et al., 2014; Zych et al., 2018). Christo et al. (2011) showed that NO plays a role in DN due to the free radical nature of NO that contribute to tubular injuries. NO encourages renal injury by accumulation of cytotoxic peroxynitrite that can induce tubular necrosis, resulting in DN (Pacher et al., 2007).

Augmentation in lipid peroxidation and NO levels leads to oxidative stress that may cause depletion in antioxidant defense enzymes. GPX, SOD, and catalases have been implicated in STZ-induced DN in previous studies (West, 2000; Sharma et al., 2006). Abovementioned data further endorsed the antioxidant potential of SA pretreatment STZ-induced DN rats. Overall, 20 and 40 mg/kg SA pretreatment of STZ-induced DN rats significantly suppressed the LPO and NO levels and replenished the normal levels of antioxidant defense enzymes in a dose-dependent scenario. The Nrf2/HO-1 pathway is a crucial cytodefense redox mechanism for dealing oxidative stress (Itoh et al., 1999a; Wang J. et al., 2015). The pathway controls intracellular phase II detoxifying enzymes to counteract ROS to encourage cellular redox steady state (Zhang, 2006). HO-1, GPx, and NAD(P)H NQO1 are upregulated by a regulatory antioxidant element in reaction to oxidative stress (Chan et al., 2001). The augmentation of serum lipids is primarily caused by upregulation in the transport of free fatty acids from adipose deposits due to lipase production. Hence, an escalation in the free lipids levels in DN (Vaziri, 2006). The pretreatment of 20 and 40 mg/kg SA on STZ-induced DN rats tended to bring the serum lipid levels close to normal levels. SA has antihyperglycemic, antihyperlipidemic, and antioxidant activity (Wilson et al., 2011; Silambarasan et al., 2014; Nithya and Subramanian, 2015; Nithya et al., 2017; Zych et al., 2018; Zych et al., 2019), which may reduce how prone lipids are to oxidation and stabilize the membrane lipids, thus inhibiting oxidative stress. Therefore, SA treatments demonstrated hypo-cholesterolemic, hypo-phospholipidemic and hypo-triglyceridemic activity, but at the same time increased HDL levels. Increases in BUN, SCr, and 24 h urea protein levels in STZ-induced DN rats are markers for the progression of DN (Makino et al., 2002). Taking account of above data SA pretreatment suggests that SA acts as a nephroprotective agent against DN or delaying its progression. Patients with diabetes have increased HO-1 protein expression due to oxidative stress. The pretreatment of STZ-induced DN rats with SA upregulated the expression of HO-1 through the activation of Nrf2 expression. Apoptosis cause to renal impairment following to ischemia in DM (Bonegio and Lieberthal, 2002; Saikumar and Venkatachalam, 2003; Vaghasiya et al., 2011; Kenan Kinaci et al., 2012).

**Figure 3:**
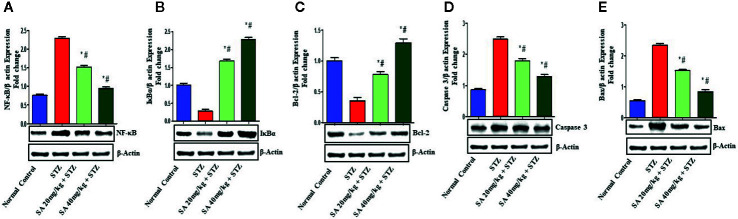
Effect of sinapic acid on the protein expression of **(A)** nuclear factor kappa B (NF-kB), **(B)** IκBα protein (IkBα), **(C)** anti-apoptotic protein BCl2, **(D)** caspase 3, and **(E)** Bax in STZ-induced diabetic rats. The reported results are presented as the mean ± SEM of six animals per group. “*” Indicates significant differences to the STZ-induced diabetic group (P < 0.05); “#” indicates significant differences to the normal control.

Bcl-2 is a key protein that controls apoptosis in cells, and upregulation of Bcl-2 expression encourage cell survival (Liang et al., 2013; He et al., 2015; Sun et al., 2015; Wang L. et al., 2015; Arai et al., 2016). Earlier reports revealed that the upregulation of the Nrf2/HO-1 proteins has an anti-apoptosis role, and that the activation of Nrf2 could induce the expression of HO-1 to enhance the expression of Bcl-2 (Ma et al., 2015). Another study found that the downregulation in the renal expressions of HO-1, Nrf2, IKBα, and BCL2 and the upregulation of NF-kB and Bax in STZ-induced diabetic rats induced DN, which is similar to the results of the present study (**Figure 3**) (Raish et al., 2016; Zhang et al., 2018; Jia et al., 2019). Histological evaluation further establishes that SA pretreatment significantly ameliorates STZ-induced DN.

## Conclusion

This study demonstrates that SA a dietary antioxidant ameliorates oxidative stress and the progression of STZ-induced DN by regulating the gene expression of SOD, GPx, and catalase and *via* the upregulation of Nrf2/HO-1 and downregulation of NF-kB signaling pathway. Overall 20 and 40 mg/kg SA pretreatment significantly and dose-dependently upregulated the expression of Nrf2, HO-1, IKBα, and Bcl-2, and downregulated the expression of NF-κB. These results suggest that the protective mechanism of SA is due to its antioxidant and inflammatory activity wherein the release of pro-inflammatory cytokines and inflammatory markers (TNFα, IL-6, and MPO) is prevented, antioxidant defense enzymes are upregulated, and lipid peroxidation and NO production as well as anti-apoptotic activity are reduced. This is potentially influenced by the control of MPO, TNF-α, IL-6, Bcl-2, and NF-kB and BaX *via* the Nrf2/HO-1 signaling pathway on STZ-induced DN. It is suggested that SA ameliorates the progression of STZ-induced DN in rats *via* NRF2/HO-1-mediated pathways. Further studies are required to better explain the underlying mechanisms.

## Data Availability Statement

All datasets generated for this study are included in the article/supplementary material.

## Ethics Statement

The study was approved by the Research Ethics Committee of the College of Pharmacy, King Saud University (Riyadh, Saudi Arabia) (Ethics Reference No. KSU-SE-20-4).

## Author Contributions

The author confirms being the sole contributor of this work and has approved it for publication.

## Conflict of Interest

The author declares that the research was conducted in the absence of any commercial or financial relationships that could be construed as a potential conflict of interest.

The reviewer MA declared a shared affiliation, with no collaboration, with the author, AA, to the handling editor at the time of review.

## References

[B1] Aebi H. (1974). “Catalase,” in Methods of enzymatic analyses. Ed. Bergmeyer H. U. (New York: Academic Press), 673–683.

[B2] Andreasen M. F. Landbo A.-K. Christensen L. P. Hansen Å. Meyer A. S. (2001). Antioxidant effects of phenolicrye (Secale cereale L.) extracts, monomeric hydroxycinnamates, and ferulic acid dehydrodimers on human low-density lipoproteins. J. Agric. Food Chem. 49, 4090–4096. 10.1021/jf0101758 11513715

[B3] Anjaneyulu M. Chopra K. (2004). Quercetin, an anti-oxidant bioflavonoid, attenuates diabetic nephropathy in rats. Clin. Exp. Pharmacol. Physiol. 31, 244–248. 10.1111/j.1440-1681.2004.03982.x 15053821

[B4] Arai S. Kitada K. Yamazaki T. Takai R. Zhang X. Tsugawa Y. (2016). Apoptosis inhibitor of macrophage protein enhances intraluminal debris clearance and ameliorates acute kidney injury in mice. Nat. Med. 22, 183–193. 10.1038/nm.4012 26726878

[B5] Bidani A. K. Picken M. Hacioglu R. Williamson G. Griffin K. A. (2007). Spontaneously reduced blood pressure load in the rat streptozotocin-induced diabetes model: potential pathogenetic relevance. Am. J. Physiol. Renal Physiol. 292, F647–F654. 10.1152/ajprenal.00017.2006 16968892 PMC1794259

[B6] Bonegio R. Lieberthal W. (2002). Role of apoptosis in the pathogenesis of acute renal failure. Curr. Opin. Nephrol. Hypertens. 11 (3), 301–308. 10.1097/00041552-200205000-00006 11981260

[B7] Brownlee M. (2005). The pathobiology of diabetic complications: a unifying mechanism. Diabetes 54, 1615–1625. 10.2337/diabetes.54.6.1615 15919781

[B8] Bryan N. S. Grisham M. B. (2007). Methods to detect nitric oxide and its metabolites in biological samples. Free Radical Biol. Med. 43 (5), 645–657. 10.1016/j.freeradbiomed.2007.04.026 17664129 PMC2041919

[B9] Chan K. Han X.-D. Kan Y. W. (2001). An important function of Nrf2 in combating oxidative stress: detoxification of acetaminophen. Proc. Natl. Acad. Sci. 98, 4611–4616. 10.1073/pnas.081082098 11287661 PMC31882

[B10] Chen Q. Peng H. Dong L. Chen L. Ma X. Peng Y. (2016). Activation of the NRF2-ARE signaling pathway by the Lentinula edodes polysaccharose LNT alleviates ROS-mediated cisplatin nephrotoxicity. Int. Immunopharmacol. 36, 1–8. 10.1016/j.intimp.2016.04.007 27093515

[B11] Chen C. (2016). Sinapic Acid and Its Derivatives as Medicine in Oxidative Stress-Induced Diseases and Aging. Oxid. Med. Cell Longev. 2016, 3571614. 10.1155/2016/3571614 27069529 PMC4812465

[B12] Cheng D. Liang B. Li Y. (2013). Antihyperglycemic effect of Ginkgo biloba extract in streptozotocin-induced diabetes in rats. BioMed. Res. Int. 2013, 162724. 10.1155/2013/162724 23509685 PMC3591163

[B13] Christo J. S. Rodrigues A. M. Mouro M. G. Cenedeze M. A. Simoes Mde J. Schor N. (2011). Nitricoxide (NO) is associated with gentamicin (GENTA) nephrotoxicity and the renal function recovery after suspension of GENTA treatment in rats. Nitric. Oxide 24, 77–83. 10.1016/j.niox.2010.12.001 21167952

[B14] Debnam E. S. Unwin R. J. (1996). Hyperglycemia and intestinal and renal glucose transport: implications for diabetic renal injury. Kidney Int. 50, 1101–1109. 10.1038/ki.1996.416 8887266

[B15] DeFronzo R. A. Bonadonna R. C. Ferrannini E. (1992). Pathogenesis of NIDDM. A balanced overview. Diabetes Care 15, 318–368. 10.2337/diacare.15.3.318 1532777

[B16] Dunlop M. (2000). Aldose reductase and the role of the polyol pathway in diabetic nephropathy. Kidney Int. 58, S3–S12. 10.1046/j.1523-1755.2000.07702.x 10997684

[B17] Firat U. Kaya S. Cim A. Buyukbayram H. Gokalp O. Dal M. S. (2012). Increased caspase-3 immunoreactivity of erythrocytes in STZ diabetic rats. Exp. Diabetes Res. 2012, 316384. 10.1155/2012/316384 22611373 PMC3350965

[B18] Flohé L. Günzler W. A. (1984). “[12] Assays of glutathione peroxidase,” in Methods in Enzymology Oxygen Radicals in Biological Systems (Academic Press), 114–120. 10.1016/S0076-6879(84)05015-1 6727659

[B19] Fridlyand L. E. Philipson L. H. (2006). Oxidative reactive species in cell injury: mechanisms in diabetes mellitus and therapeutic approaches. Ann. New York Acad. Sci. 1066, 136–151. 10.1196/annals.1363.019 16533924

[B20] Gheith O. Farouk N. Nampoory N. Halim M. A. Al-Otaibi T. (2016). Diabetic kidney disease: world wide difference of prevalence and risk factors. J. Nephropharmacol. 5, 49–56. 10.4103/1110-9165.197379 28197499 PMC5297507

[B21] Giacco F. Brownlee M. (2010). Oxidative stress and diabetic complications. Circ. Res. 107, 1058–1070. 10.1161/CIRCRESAHA.110.223545 21030723 PMC2996922

[B22] Giri B. Dey S. Das T. Sarkar M. Banerjee J. Dash S. K. (2018). Chronic hyperglycemia mediated physiological alteration and metabolic distortion leads to organ dysfunction, infection, cancer progression and other pathophysiological consequences: An update on glucose toxicity. BioMed. Pharmacother. 107, 306–328. 10.1016/j.biopha.2018.07.157 30098549

[B23] He X. Xie Z. Dong Q. Li J. Li W. Chen P. (2015). Effect of Folic Acid Supplementation on Renal Phenotype and Epigenotype in Early Weanling Intrauterine Growth Retarded Rats. Kidney Blood Press Res. 40, 395–402. 10.1159/000368516 26202812

[B24] Hoybergs Y. M. Biermans R. L. Meert T. F. (2008). The impact of bodyweight and body condition on behavioral testing for painful diabetic neuropathy in the streptozotocin rat model. Neurosci. Lett. 436, 13–18. 10.1016/j.neulet.2008.02.051 18375060

[B25] Itoh K. Ishii T. Wakabayashi N. Yamamoto M. (1999a). Regulatory mechanisms of cellular response to oxidative stress. Free Radical Res. 31, 319–324. 10.1080/10715769900300881 10517536

[B26] Itoh K. Wakabayashi N. Katoh Y. Ishii T. Igarashi K. Engel J. D. (1999b). Keap1 represses nuclear activation of antioxidant responsive elements by Nrf2 through binding to the amino-terminal Neh2domain. Genes Dev. 13, 76–86. 10.1101/gad.13.1.76 9887101 PMC316370

[B27] Jeong S.-O. Oh G.-S. Ha H.-Y. Soon Koo B. Sung Kim H. Kim Y.-C. (2009). Dimethoxycurcumin, a synthetic curcumin analogue, induceshemeoxygenase-1 expression through Nrf2 activation in RAW264. 7 macrophages. J. Clin. Biochem. Nutr. 44, 79–84. 10.3164/jcbn.08-194 19177192 PMC2613503

[B28] Jia Q. Yang R. Liu X. F. Ma S. F. Wang L. (2019). Genistein attenuates renal fibrosis in streptozotocin induced diabetic rats. Mol. Med. Rep. 19, 423–431. 10.3892/mmr.2018.9635 30431100 PMC6297769

[B29] Kanchana G. Shyni W. J. Malini P. Rajadurai M. (2011). Effect of Sinapic acid on antiperoxidative and antioxidant potential in normal and Streptozotocin-induced diabetes in Wistar rats. IJPCR 3, 5–9.

[B30] Karthik D. (2014). Insilico docking analysis of Dipeptidylpeptidase-4 (DPP-IV or CD26) with some selective bioflavonoids using Genetic Lamarckian Algorithm. J. Comput. Methods Mol. Des. 4, 24–31.

[B31] Kenan Kinaci M. Erkasap N. Kucuk A. Koken T. Tosun M. (2012). Effects of quercetin on apoptosis, NF-κB and NOS gene expression in renal ischemia/reperfusion injury. Exp. Ther. Med. 3, 249–254. 10.3892/etm.2011.382 22969877 PMC3438559

[B32] Kiritoshi S. Nishikawa T. Sonoda K. Kukidome D. Senokuchi T. Matsuo T. (2003). Reactive Oxygen Species from Mitochondria Induce Cyclooxygenase-2 Gene Expression in Human Mesangial Cells Potential Role in Diabetic Nephropathy. Diabetes 52, 2570–2577. 10.2337/diabetes.52.10.2570 14514642

[B33] Klein G. Kim J. Himmeldirk K. Cao Y. Chen X. (2007). Antidiabetes and Anti-obesity Activity of Lagerstroemia speciosa. Evid. Based Complement. Alternat. Med. 4, 401–407. 10.1093/ecam/nem013 18227906 PMC2176148

[B34] Kundu A. Dey P. Sarkar P. Karmakar S. Tae I. H. Kim K. S. (2020). Protective effects of Croton hookerion streptozotocin-induced diabetic nephropathy. Food Chem. Toxicol. 135, 110873. 10.1016/j.fct.2019.110873 31600566

[B35] Liang H. Yu F. Tong Z. Yuan B. Wang C. (2013). Effect of ischemia post-conditioning on skeletal muscle oxidative injury, mTOR, Bax, Bcl-2 proteins expression, and HIF-1α/β-actin mRNA, IL-6/β-actin mRNA and caveolin-3/β-actin mRNA expression in ischemia-reperfusion rabbits. Mol. Biol. Rep. 40, 507–514. 10.1007/s11033-012-2087-9 23108993

[B36] Lowry O. H. Rosebrough N. J. Farr A. L. Randall R. J. (1951). Protein measurement with the Folin phenol reagent. J. Biol. Chem. 193, 265–275. 14907713

[B37] Ma W. Hu J. Cheng Y. Wang J. Zhang X. Xu M. (2015). Ginkgolide b protects against cisplatin-induced ototoxicity: Enhancement of akt-nrf2-ho-1 signaling and reduction of nadph oxidase. Cancer Chemother. Pharmacol. 75, 949–959. 10.1007/s00280-015-2716-9 25749575

[B38] Makino H. Tanaka I. Mukoyama M. Sugawara A. Mori K. Muro S. (2002). Prevention of diabetic nephropathy in rats by prostaglandin E receptorEP1-selective antagonist. J. Am. Soc. Nephrol. 13, 1757–1765. 10.1097/01.ASN.0000019782.37851.BF 12089371

[B39] Meo S. A. Memon A. N. Sheikh S. A. Rouq F. A. Usmani A. M. Hassan A. (2015). Effect of environmental air pollutionon type 2 diabetes mellitus. Eur. Rev. Med. Pharmacol. Sci. 19, 123–128. 25635985

[B40] Mestry S. N. Dhodi J. B. Kumbhar S. B. Juvekar A. R. (2017). Attenuation of diabetic nephropathy in streptozotocin-induced diabetic rats by Punica granatum Linn. leaves extract. J. Tradit. Complement. Med. 7, 273–280. 10.1016/j.jtcme.2016.06.008 28725620 PMC5506633

[B41] Nezu M. Suzuki N. Yamamoto M. (2017). Targeting the KEAP1-NRF2 System to Prevent Kidney Disease Progression. Am. J. Nephrol. 45, 473–483. 10.1159/000475890 28502971

[B42] Nguyen T. Sherratt P. J. Huang H. C. Yang C. S. Pickett C. B. (2003). Increased protein stability as a mechanism that enhances Nrf2-mediated transcriptional activation of the antioxidant response element. Degradation of Nrf2 by the 26S proteasome. J. Biol. Chem. 278, 4536–4541. 10.1074/jbc.M207293200 12446695

[B43] Nićiforović N. Abramovič H. (2014). Sinapic acid and its derivatives: natural sources and bioactivity. Compr. Rev. Food Sci. Food Saf. 13, 34–51. 10.1111/1541-4337.12041 33412688

[B44] Niehaus W. Jr. Samuelsson B. (1968). Formation of malonaldehyde from phospholipid arachidonate during microsomal lipid peroxidation. Eur. J. Biochem. 6 (1), 126–130. 10.1111/j.1432-1033.1968.tb00428.x 4387188

[B45] Nishikawa T. Edelstein D. Du X. L. Yamagishi S. Matsumura T. Kaneda Y. (2000). Normalizing mitochondrial superoxide production blocks three pathways of hyperglycaemic damage. Nature 404, 787–790. 10.1038/35008121 10783895

[B46] Nithya R. Subramanian S. (2015). Sinapic Acid, a Naturally Occurring Carboxylic Acid Derivative Ameliorates Hyperglycemia in High Fat Diet-Low Dose STZ Induced Experimental Diabetic Rats. Int. J. Sci. Eng. Technol. Res. 4, 5746–5750.

[B47] Nithya R. Devi V. Selvam R. Subramanian S. (2017). Sinapic acid regulates glucose homeostasis by modulating the activities of carbohydrate metabolizing enzymes in high fat diet fed-low dose STZ induced experimental type 2 diabetes in rats. Global J. Obes. Diabetes Metab. Syndr. 4, 54–61.

[B48] Ohkawa H. Ohishi N. Yagi K. (1979). Assay for lipid peroxides in animal tissues by thiobarbituric acid reaction. J. Anal. Biochem. 95 (2), 351–358. 10.1016/0003-2697(79)90738-3 36810

[B49] Pacher P. Beckman J. S. Liaudet L. (2007). Nitric oxide and peroxynitrite in health and disease. Physiol. Rev. 87, 315–424. 10.1152/physrev.00029.2006 17237348 PMC2248324

[B50] Pan H.-Z. Zhang L. Guo M.-Y. Sui H. Li H. Wu W.-H. (2010). Theoxidative stress status in diabetes mellitus and diabetic nephropathy. Acta Diabetol. 47, 71–76. 10.1007/s00592-009-0128-1 19475334

[B51] Raish M. Ahmad A. Jan B. L. Alkharfy K. M. Ansari M. A. Mohsin K. (2016). Momordica charantia polysaccharides mitigate the progression of STZ induced diabetic nephropathy in rats. Int. J. Biol. Macromol. 91, 394–399. 10.1016/j.ijbiomac.2016.05.090 27238589

[B52] Ray P. D. Huang B. W. Tsuji Y. (2012). Reactive oxygen species(ROS) homeostasis and redox regulation in cellular signaling. Cell Signal 24, 981–990. 10.1016/j.cellsig.2012.01.008 22286106 PMC3454471

[B53] Ricardo S. D. Bertram J. F. Ryan G. B. (1994). Reactive oxygen species in puromycin aminonucleoside nephrosis: in vitro studies. Kidney Int. 45, 1057–1069. 10.1038/ki.1994.142 8007575

[B54] Saikumar P. Venkatachalam M. A. (2003). Role of apoptosis in hypoxic/ischemic damage in the kidney. Semin. Nephrol. 23, 511–521. 10.1053/S0270-9295(03)00130-X 14631559

[B55] Satheesh G. Ramachandran S. Jaleel A. (2020). Metabolomics-Based Prospective Studies and Prediction of Type 2 Diabetes Mellitus Risks. Metab. Syndr. Relat. Disord. 18, 1–9. 10.1089/met.2019.0047 31634052

[B56] Scheen A. J. (1997). Drug treatment of non-insulin-dependent diabetes mellitus in the 1990s. Achievements and future developments. Drugs 54, 355–368. 10.2165/00003495-199754030-00001 9279500

[B57] Sharma S. Kulkarni S. K. Chopra K. (2006). Curcumin, the active principle of turmeric (Curcuma longa), ameliorates diabetic nephropathy in rats. Clin. Exp. Pharmacol. Physiol. 33, 940–945. 10.1111/j.1440-1681.2006.04468.x 17002671

[B58] Shen X. Hu B. Xu G. Chen F. Ma R. Zhang N. (2017). Activation of Nrf2/HO-1 Pathway by Glycogen Synthase Kinase-3β Inhibition Attenuates Renal Ischemia/Reperfusion Injury in Diabetic Rats. KBR 42, 369–378. 10.1159/000477947 28624830

[B59] Sharma I. Aaradhya M. Kodikonda M. Naik P. R. (2015). Antihyperglycemic, antihyperlipidemic and antioxidant activity of phenolic rich extract of Brassica oleraceae var gongylodes on streptozotocin induced Wistar rats. Springer plus 4, 212. 10.1186/s40064-015-0948-0 PMC4439412 26020019

[B60] Shokeen P. Anand P. Murali Y. K. Tandon V. (2008). Antidiabetic activity of 50% ethanolic extract of Ricinus communis and its purified fractions. Food Chem. Toxicol. 46, 3458–3466. 10.1016/j.fct.2008.08.020 18790711

[B61] Silambarasan T. Manivannan J. Krishna Priya M. Suganya N. Chatterjee S. Raja B. (2014). Sinapic acid prevents hypertension and cardiovascular remodeling in pharmacological model of nitric oxide inhibited rats. PloS One 9, e115682. 10.1371/journal.pone.0115682 25531679 PMC4274097

[B62] Silambarasan T. Manivannan J. Raja B. Chatterjee S. (2016). Prevention of cardiac dysfunction, kidney fibrosis and lipid metabolic alterations in l-NAME hypertensive rats by sinapic acid-Role of HMG-CoA reductase. Eur. J. Pharmacol. 777, 113–123. 10.1016/j.ejphar.2016.03.004 26945821

[B63] Spiller H. A. Sawyer T. S. (2006). Toxicology of oral antidiabetic medications. Am. J. Health Syst. Pharm. 63, 929–938. 10.2146/ajhp050500 16675650

[B64] Srivastava S. Shree P. Pandey H. Tripathi Y. B. (2018). Incretin hormones receptor signaling plays the key role in antidiabetic potential of PTY-2 against STZ-induced pancreatitis. Biomed. Pharmacother. 97, 330–338. 10.1016/j.biopha.2017.10.071 29091882

[B65] Stenvinkel P. Painer J. Kuro O. M. Lanaspa M. Arnold W. Ruf T. (2018). Novel treatment strategies for chronic kidney disease: insights from the animal kingdom. Nat. Rev. Nephrol. 14, 265–284. 10.1038/nrneph.2017.169 29332935

[B66] Sugimoto K. I. Tsuruoka S. Fujimura A. (2001). Effect of Enalapril On Diabetic Nephropathy In Oletf Rats: The Role Of An Anti-Oxidative Action In Its Protective Properties. Clin. Exp. Pharmacol. Physiol. 28, 826–830. 10.1046/j.1440-1681.2001.03530.x 11553023

[B67] Sun Y. Oberley L. W. Li Y. (1988). A simple method for clinical assay of superoxide dismutase. Clin. Chem. 34 (3), 497–500. 10.1093/clinchem/34.3.497 3349599

[B68] Sun Y. Zhang T. Li L. Wang J. (2015). Induction of apoptosis by hypertension via endoplasmic reticulum stress. Kidney Blood Press Res. 40, 41–51. 10.1159/000368481 25791362

[B69] Sun W. Liu X. Zhang H. Song Y. Li T. Liu X. (2017). Epigallocatechin gallate upregulates NRF2 to prevent diabetic nephropathy via disabling KEAP1. Free Radic. Biol. Med. 108, 840–857. 10.1016/j.freeradbiomed.2017.04.365 28457936

[B70] Tan A. L. Forbes J. M. Cooper M. E. (2007). AGE, RAGE, and ROS in diabetic nephropathy. Semin. Nephrol. 27, 130–143. 10.1016/j.semnephrol.2007.01.006 17418682

[B71] Tikoo K. Bhatt D. K. Gaikwad A. B. Sharma V. Kabra D. G. (2007a). Differential effects of tannic acid on cisplatin induced nephrotoxicity in rats. FEBS Lett. 581, 2027–2035. 10.1016/j.febslet.2007.04.036 17470369

[B72] Tikoo K. Tripathi D. N. Kabra D. G. Sharma V. Gaikwad A. B. (2007b). Intermittent fasting prevents the progression of type I diabetic nephropathy in rats and changes the expression of Sir2 and p53. FEBS Lett. 581, 1071–1078. 10.1016/j.febslet.2007.02.006 17316625

[B73] Towbin H. Staehelin T. Gordon J. (1979). Electrophoretic transfer of proteins from polyacrylamide gels to nitrocellulose sheets: procedure and some applications. Proc. Natl. Acad. Sci. U.S.A. 76, 4350–4354. 10.1073/pnas.76.9.4350 388439 PMC411572

[B74] Vaghasiya J. Sheth N. Bhalodia Y. Manek R. (2011). Sitagliptin protects renal ischemia reperfusion induced renal damage in diabetes. Regul. Peptides 166, 48–54. 10.1016/j.regpep.2010.08.007 20728477

[B75] Vaziri N. (2006). Dyslipidemia of chronic renal failure: the nature, mechanisms, and potential consequences. Am. J. Physiol.-Renal Physiol. 290, F262–F272. 10.1152/ajprenal.00099.2005 16403839

[B76] Wang L. Liu X. Chen H. Chen Z. Weng X. Qiu T. (2015). Effect of picroside II on apoptosis induced by renal ischemia/reperfusion injury in rats. Exp. Ther. Med. 9, 817–822. 10.3892/etm.2015.2192 25667634 PMC4316970

[B77] Wang J. Hu X. Xie J. Xu W. Jiang H. (2015). Beta-1-adrenergic receptors mediate Nrf2-HO-1-HMGB1 axis regulation to attenuate hypoxia/reoxygenation-induced cardiomyocytes injury in vitro. Cell Physiol. Biochem. 35, 767–777. 10.1159/000369736 25634756

[B78] Wee A. (1991). A practical approach to the liver biopsy. Malays. J. Pathol. 13, 75–88. 1823095

[B79] West I. C. (2000). Radicals and oxidative stress in diabetes. Diabet. Med. 17, 171–180. 10.1046/j.1464-5491.2000.00259.x 10784220

[B80] Wilson J. S. Ganesan K. Palanisamy M. J. (2011). Effect of sinapic acid on biochemical markers and histopathological studies in normal and streptozotocin-induced diabetes in wistar rats. Int. J. Pharm. Pharm. Sci. 3 (4 Suppl), 115–120.

[B81] Xie Z. Zhong L. Wu Y. Wan X. Yang H. Xu X. (2018). Carnosic acid improves diabetic nephropathy by activating Nrf2/ARE and inhibition of NF-kappa B pathway. Phytomedicine 47, 161–173. 10.1016/j.phymed.2018.04.031 30166101

[B82] Zhang L. Chen Z. Gong W. Zou Y. Xu F. Chen L. (2018). Paeonol Ameliorates Diabetic Renal Fibrosis Through Promoting the Activation of the Nrf2/ARE Pathway via Up-Regulating Sirt1. Front. Pharmacol. 9, 512. 10.3389/fphar.2018.00512 29867511 PMC5968333

[B83] Zhang D. D. (2006). Mechanistic studies of the Nrf2-Keap1 signaling pathway. Drug Metab. Rev. 38, 769–789. 10.1080/03602530600971974 17145701

[B84] Zych M. Kaczmarczyk-Sedlak I. Wojnar W. Folwarczna J. (2018). The Effects of Sinapic Acid on the Development of Metabolic Disorders Induced by Estrogen Deficiency in Rats. Oxid. Med. Cell Longev. 2018, 9274246. 10.1155/2018/9274246 29967666 PMC6008867

[B85] Zych M. Wojnar W. Borymski S. Szalabska K. Bramora P. Kaczmarczyk-Sedlak I. (2019). Effect of Rosmarinic Acid and Sinapic Acid on Oxidative Stress Parameters in the Cardiac Tissue and Serum of Type 2 Diabetic Female Rats. Antioxidants 8, 579. 10.3390/antiox8120579 PMC6943504 31771099

